# Monitoring of long-lasting insecticidal nets (LLINs) coverage versus utilization: a community-based survey in malaria endemic villages of Central India

**DOI:** 10.1186/s12936-017-2117-0

**Published:** 2017-11-17

**Authors:** Kamaraju Raghavendra, Mehul Kumar Chourasia, Dipak Kumar Swain, Rajendra M. Bhatt, Sreehari Uragayala, G. D. P. Dutta, Immo Kleinschmidt

**Affiliations:** 1 National Institute of Malaria Research (ICMR), Sector-8, Dwarka, New Delhi, 110077 India; 2 National Institute of Malaria Research (ICMR) IIR-WHO Project, Field Unit, Kondagaon, Chhattisgarh India; 3 National Institute of Malaria Research (ICMR), Field Unit, Lalpur, Raipur, Chhattisgarh India; 4 National Institute of Malaria Research (ICMR), Field Unit, Bangalore, Karnataka India; 5 Department of Infectious Disease Epidemiology, London School of Hygiene and Tropical Medicine, London, UK

**Keywords:** Long-lasting insecticidal nets (LLINs), LLINs uses, LLINs coverage, Chhattisgarh, India

## Abstract

**Background:**

Despite the known effectiveness of long-lasting insecticidal nets (LLINs) in providing protection against malaria, high level of ownership and use are very difficult to achieve and maintain. Nearly 40,000 LLINs were distributed in 2014 as an intervention tool against malaria transmission in 80 villages of Keshkal sub-district in Chhattisgarh, India. This study assessed LLIN coverage, access, utilization pattern, and key determinants for the net use 1 year after mass distribution.

**Methods:**

In 2015, a cross-sectional household survey was carried out in 80 study clusters (whole village or part of village). From each cluster, 40 households were randomly selected and interviewed using a structured questionnaire adapted from the malaria indicator survey of Roll Back Malaria guidelines. Information on demographic characteristics, LLIN ownership, and its use on the night before the survey, and physical condition of LLINs were recorded.

**Results:**

2970 households were interviewed with a total of 15,003 individuals present in the households during the night before the survey. Nearly 98% of households had at least one LLIN and 59.4% of the surveyed population reportedly used an LLIN the previous night. LLIN use varied from 41 to 94% between the study clusters. Nearly 89% of the LLINs were found in good physical condition (without holes). However, proportion of household with at least one LLIN per two persons was only 39%.

**Conclusion:**

Universal coverage of LLINs was inadequate in the study clusters making it difficult for all household members to use an LLIN. LLIN use varied between clusters and was highest in children under 5 years of age. Health education campaigns and creating awareness about the benefit of sleeping under the LLINs in providing protection against malaria is required not only to high risk groups of pregnant women and children below 5 years of age but all the members of the family to have an epidemiological impact of this intervention at the community level. Relatively high net use despite poor access to LLINs indicates an overall desire to use nets when they are available. The main barrier to increased use of nets is the low coverage at household level.

## Background

Use of long-lasting insecticidal nets (LLINs) is a widely implemented and cost-effective public health intervention tool for malaria control and prevention in most malaria endemic countries [1–3]. Systematic review suggests that universal LLIN coverage with adequate usage may reduce the incidence of clinical malaria by up to 50% in malaria endemic (stable) areas [4]. Among children under 5 years of age, LLINs provide up to 55% protective efficacy in preventing malaria attributed mortality [5, 6]. Operational success can only be achieved when universal coverage is attained and at least 80% of bed nets are actually used by the community [7]. However, the impact on reduction of malaria varies by underlying transmission intensity and has not been established.

LLIN mass distribution campaigns and supplementary distribution through antenatal clinics and immunization services have largely been successful in achieving increased LLIN ownership and universal LLIN coverage in the population [8–13]. Several African studies have shown an apparent difference in adequate LLIN possession (one LLIN per two person) and LLIN use by the community [14–18]. Therefore, routine monitoring for LLIN use and regular community-tailored education and awareness campaigns may be vital to sustain the utilization and to optimize the protective effect of LLINs [12, 19].

In the past two decades, a number of studies, mostly from African continent, have explored various factors and determinants for decline in bed net use in the household as well as at the community level. The major reported factors for non-use were inadequate net availability, heat and discomfort due to sleeping under the net, absence of mosquito nuisance, seasonal variation, sleeping pattern and preferences for net uses among household members [16, 18, 20–23].

Since 2009, India has initiated scaling up of LLIN interventions under the National Vector Borne Disease Control Programme (NVBDCP) to control malaria in endemic states [24]. In 2016, an action plan for malaria elimination by 2030 has emphasized LLIN interventions and indoor residual spraying (IRS) of insecticide. Monitoring of LLIN access and use is needed at household and community levels to optimize the impact of LLIN based intervention and sustain effective malaria control in the region.

There is scant information on large-scale community based assessment studies on LLIN coverage and usage pattern post mass LLIN distribution under the national programme from the Indian sub-continent [25, 26]. Limited studies have explored the factors which affect LLIN access and use and reasons for attrition in the Indian population especially among tribal inhabitants [27].

The main objective of the present study was to evaluate LLIN ownership, access, use and attrition rate in the tribal population in 80 study clusters (whole village or part of village) of Keshkal sub-district, Chhattisgarh, India, 1 year after mass distribution of LLIN. This study also investigated the factors affecting the LLIN use and reasons for non-use in the study population. This study was implemented as part of the research project on Implications of insecticide resistance (IIR) on malaria vector control [28].

## Methods

### Study area and study design

A clustered-sample, cross-sectional household survey was undertaken during August–November 2015, 1 year after mass LLIN distribution. The study was carried out in 80 study clusters of Keshkal (20°5′1 N and 81°35′12 E), a sub-district in Kondagaon district in Chhattisgarh state, India. Study villages are predominantly inhabited by the ‘*Gond*’ tribe and basic livelihood depends on subsistence agriculture, collection and selling of forest products, and manual labour. The major crop is paddy, cultivated during the monsoon season, starting from late-June to mid-October. Main malaria transmission season in the region overlaps with the cultivating season. *Anopheles culicifacies* is the primary vector of malaria in the study area.

### LLINs distribution

PermaNet 2.0, a deltamethrin (55 mg/m^2^) impregnated polyester LLIN, manufactured by Vestergaard Frandsen (Switzerland) were provided by the State Health Department for distribution in the study villages. A census of all the households was done in 2013. Based on census, household list with total family members was prepared and then number of nets a household would receive was determined. Project staff and field workers were trained for LLIN distribution and health education. In most of the villages, nets were distributed through a fixed point approach in *Anganwadi* centres (Children day care centres), schools, or *Gram Panchayat* (village council) buildings. All households were informed about LLIN distribution in their respective villages. However, if any household was missed during the distribution round due to the house being locked or due to the absence of adult members, additional mop-up rounds of distribution were made through door-to-door visits to ensure that no household was left without a net. By the end of distribution during November–December 2014, about 40,000 nets were distributed covering ~ 80,000 of population in all the villages. At the time of net distribution, a printed handbill in the local language on use and care of LLINs was provided. The content was also read to householders at opportune times during the follow up visits by the project personnel. High turnout of the community for receiving the nets was recorded. Based on census records, 99.5% LLIN coverage was achieved barring inhabitants in locked houses despite mopping and an average of two LLINs [95% confidence interval (CI) = 1.7–2.3] per household was achieved. Outer polythene covers of LLINs were retained and along with the packing (HDPE sacs) material were sent for incineration in the bio-medical waste management plant to prevent environmental pollution and hazard.

Distribution of LLINs was done as per the national guidelines, i.e., 1 net for 2.5 persons [29] which was changed to 1 net per 2 persons [30]. Due to these revised criteria, the calculated LLIN coverage was reduced to 40.9% which created a shortfall of about 10,000 nets. Efforts made to procure the additional quantity of nets to improve the coverage were not achieved.

### Study questionnaires and variables

A structured questionnaire adapted from Malaria Indicator Survey (2013) as per Roll Back Malaria (RBM) guidelines was used for evaluating compliance of LLIN among the individuals in the households and the effectiveness of the distribution [31]. The questionnaire consisted of three parts: general information; individual net access and compliance to LLIN use; and LLIN’s physical integrity. The questionnaire was translated into Hindi and back translated into English to verify the validity of the translation. The questionnaire was pre-tested and piloted to identify any errors or misinterpretation due to wording or the translation.

### Study variable definitions and indicators

The main outcome of interest was ‘last night LLIN use’, defined as the proportion of residents who reported to have slept under an LLIN the night preceding the survey. The following indicators were collected: (1) proportion of households with at least one LLIN; (2) proportion of households with at least one LLIN for every two people; (3) proportion of existing LLINs used the previous night; (4) proportion of population that slept under a LLIN the previous night; (5) proportion of children under 5 years old who slept under a LLIN the previous night. For this study, member of one household was defined as ‘all the members residing under one roof and sharing a common cooking place’.

### Sample size calculation

Two-stage cluster sampling design with ‘household’ as primary sampling units (PSUs) was used to select sample in the survey. Cluster level sampling frame was used to prepare the list of all the selected households. Furthermore, 40 households were sampled from each study cluster which were identified by systematic random sampling. A sample size was calculated for the precision of 5 and 50% of expected point estimate of the proportion of community residents sleeping under LLINs in the previous night, 95% confidence interval with assumption of 90% of response and design effect of 1.8. Hence, a total of 3200 households from 80 study clusters were sampled during the survey.

### Study data collection

Based on selected list, households were approached for the survey. The survey was carried out by skilled and trained project staff after a day of orientation and field training for conducting the activity. The interview procedure was described to the respondent before the start of the interview. Where a selected house was found locked, the next house was used as a replacement. Interviews were conducted within the premises of the respondent’s house using Hindi or local language (Chhattisgarhi and Halbi dialects) for ease of communication.

### Data entry and statistical analysis

All the data were entered twice in EpiData version 3 and statistical analysis was carried out in SPSS 20 version (IBM statistics, NY). Continuous variables were expressed as mean and standard deviation (SD) while categorical variables were expressed as number and proportion. The association between the outcome and independent variables was assessed using generalized estimating equations (GEE) to allow for clustering at village level. Study clusters were taken as subjects. ‘LLIN use’ was response variable in the model. Binomial distribution with logit link function and exchangeable correlation structure was selected for the GEE model. Study cluster variable was taken at the subject level. Firstly, in the univariate GEE model, all possible exposure variables such as literacy of head of the households, type of house, household size, and age of family members were included in the model. Then, in the final multivariate GEE model, all those variables that were significant in the univariate model were included. Tables were prepared with unadjusted and adjusted odds ratios (95% confidence intervals) and respective p value (Table 3).

### Ethical clearance and informed consent

Informed consent was obtained from the head of households to participate in the study at the time of LLINs distribution and during the data collection. This study was undertaken as a part of a WHO-coordinated multi-country project and ethical clearance was obtained from the Institutional Ethics Committee of National Institute of Malaria Research, India (ECR/NIMR/EC/2010/75).

## Results

### Socio-demographic characteristics of study population

A total of 2970 households with average household size (standard deviation) of 5.3 ± 1.96 persons were surveyed in 80 clusters. In the surveyed households, the mean age of head of the households was 48.9 years. Most of them were males (83.5%) and nearly two-thirds had attended more than primary level of school (67.1%). Among the surveyed households, in majority **(**65.4%) of the houses, roofs were made up of clay tiles/tin/asbestos (Table 1).

**Table 1:** Socio-demographic characteristics of households (n = 2970) and study participants (n = 15,800)

Variable name	Variable category	Frequency, n (%)
Age of head of household (in years)	Mean (± SD)	48.9 (± 12.8)
Gender of head of household	Male	2479 ( 83.5)
	Female	491 (16.5)
Highest educational level of head of household	None	978 (32.9)
	Primary school	1390 (46.8)
	Middle school	351 (11.8)
	Secondary and higher	251 (8.5)
Household size	Mean (± SD)	5.3 (± 1.9)
Type of house (roof)	Hut/thatched	239 (8.0)
	Clay tile/tin/asbestos	1942 (65.4)
	Pakka/RCC roof	789 (26.6)
Age of household members (years)	< 5	1309 (8.3)
	5–14	2951 (18.7)
	> 14	11,540 (73.0)
Gender of household members	Male	7712 (48.8)
	Female	8808 (51.2)
Household members present last night	Yes	15,003 (94.9)
	No	797 (5.0)

The survey covered 15,800 residents with an average age of 27.7 years (SD ± 18.3). Proportion of children under 5 years of age was 8.3%, while female population contributed 51.2%.

### Net availability, access, type of bed nets and use

Overall, 98.4% of surveyed households had at least one LLIN. 80% of households were in possession of two or more LLINs (79.7%). In all, 5953 LLINs and 1730 untreated nets were present and physically observed in the surveyed houses during the survey. Only 38.7% of the household met the universal coverage criterion of 1 LLIN per two persons (Table 2).

**Table 2:** Net availability, access, type of bed nets, uses and attrition (n = 2970)

Variable name	Category	Frequency, n (%)
Households with at least one mosquito net	Yes	2942 (99.1)
LLIN ownership (households with at least one LLIN)	Yes	2923 (98.4)
Number of LLINs ownership per HH	1	602 (20.3)
	2	1668 (56.2)
	3	602 (20.3)
	≥ 4	51 (1.7)
Total number of bed nets available in the surveyed HH (n = 7683)	LLIN	5953 (77.5)
	Untreated net	1730 (22.5)
Number of LLINs per HH	Mean (± SD)	2.0 (± 0.74)
Households with at least one LLIN for every two persons	Yes	1148 (38.7)
Last night bed net use by HH members	LLIN users	9388 (59.4)
	Untreated net users	1156 (7.3)
Use of LLINs the previous night by age group	< 5 years	1018 (81.2)
	5–14 years	1853 (69.8)
	>14 years	6517 (61.8)
Use of LLINs the previous night by age group by gender	Male	4479 (63.9)
	Female	4909 (65.9)
LLIN with at least one hole (of any size)	Yes	659/5953 (11.2)
Use to access ratio (%)		59.4/98.4 (60.4)

Altogether, 59.4% of the population was reportedly using an LLIN the night before the survey, while 7.3% were using untreated nets. LLIN use was high among children under 5 years (81.2%). Among all physically observed LLINs, 11.2% had at least one hole (of any size).

### Determinants associated with LLIN use in the study population

Based on a univariate GEE model, strong to moderate associations were found between LLIN use and socio-demographic variables such as literacy of head of the households [odds ratio (OR) = 1.28, 95% confidence interval (CI) = 1.1–1.5; p = 0.001], type of house (clay tile vs hut: OR = 1.71, 95% CI 1.3–2.3; p < 0.001) household size (1–2 vs > 8: OR = 1.51, 95% CI 1.1–2.1; p = 0.021) and age of family members (2.67, 2.3–3.1; < 0.001). Additionally, a weak association was found between LLIN use and gender of household members (male vs female: OR = 1.1, 95% CI 1.01–1.2; p = 0.014). In the final multivariate GEE model, all the five explanatory variables including literacy of head of the households, type of house, age and gender of family members and persons per LLIN showed significant association with LLIN use (Table 3).

**Table 3:** Determinants associated with LLIN use among study population from households with ≥ 1 LLIN (n = 14,455) per house

Parameters	Category	Last night LLIN use n (%)	Total	Unadjusted odds ratio (95% CI)	p value	Adjusted OR (95% CI)	p value
Literacy of head of household	No	2929 (61.1)	4790	1	–	–	–
	Yes	6459 (66.8)	9665	1.28 (1.1–1.5)	0.001	1.2 (1.04–1.4)	0.012
Age group of HH members (in years)	> 14	6517 (61.8)	10,546	1	–	1	–
	5–14	1853 (69.8)	2655	1.43 (1.3–1.6)	< 0.001	1.5 (1.4–1.7)	< 0.001
	< 5	1018 (81.2)	1254	2.67 (2.3–3.1)	< 0.001	3.18 (2.7–3.7)	< 0.001
Gender of HH members	Male	4479 (63.9)	7007	1	–	1	–
	Female	4909 (65.9)	7448	1.1 (1.01–1.2)	0.014	1.1 (1.03–1.2)	0.005
Type of house	Hut/thatched	665 (54.1)	1230	1	–	1	–
	Clay tile/tin/asbestos	6231 (66.8)	9327	1.71 (1.3–2.3)	< 0.001	1.09 (0.96–1.2)	0.209
	Pakka/RCC roof	2492 (63.9)	3898	1.51 (1.1–2.0)	0.006	0.67 (0.52–0.94)	0.012
Number of person per LLIN	> 4	481 (42.9)	639	1	–	1	–
	2.1–4	5794 (64.8)	8935	2.45 (2.0–3.0)	< 0.001	2.65 (2.15–3.28)	< 0.001
	≤ 2	3113 (70.8)	4400	3.21 (2.58–4.01)	< 0.001	3.75 (2.98–4.72)	< 0.001

Subject level-study cluster (n = 80)

### Reasons cited by the non-users for non-use of LLINs

Among the total de facto population, 27.2% reported not to have used a net the night before the survey. Among non-users, 46% of the persons cited non-availability of LLIN as a major reason for not using LLIN followed by seasonal use of LLIN (7.5%). Nearly, 14% of non-users reported other means of protection such as fire, smoke, use of fan, etc. and nearly 7% perceived less mosquito density in the houses (Table 4).

**Table 4:** Various reasons cited for non-use of LLINs by non-users (n = 3937)

Sl. no	Non-use category	Major reported reasons	Frequency, n (%)
1.	Technical	Inadequate space	43 (1.1)
		No place to hang the net	12 (0.3)
		Home maintenance/painting	133 (3.4)
2.	Discomfort	Heat	26 (0.7)
		Feel closed in	44 (1.1)
		Non-habitual	251 (6.4)
		Inconvenience	5 (0.1)
3.	Non-availability	Net not available	1813 (46.1)
		Net washed	125 (3.2)
		Net given away	08 (0.2)
		Net was torn/worn out	49 (1.2)
4.	Social	Slept elsewhere	30 (0.8)
		Due to guest at home	9 (0.2)
5.	Perceived mosquito density/malaria	Less mosquito density	268 (6.8)
		No malaria now	9 (0.2)
6.	Use of mosquito control methods	Using fire/smoke	129 (3.3)
		Using fans	77 (2)
		Using blankets	292 (7.4)
		Using coil/mosquito repellent	6 (0.2)
7.	Other	Use only during rainy season	296 (7.5)
		Different use of LLIN	48 (1.2)
		Do not know	150 (3.8)
		Other	114 (2.9)

## Discussion

This study showed that 98.4% of the surveyed household had access to one LLINs and 59.4% of the residents were using LLINs during the night before the survey. A high number of LLIN uses (81.2%) among vulnerable age group of children < 5 years of age was observed, followed by 69.8% among 5–14 years of children. Similar LLIN use among high risk group in the same population has been previously observed [32]. Overall LLIN use among adults was lower than among children. This was mainly due to inadequate number of nets per household and the high focus on vulnerable age groups in the educational and awareness camps in the national malaria control programs. One other thing that is worth emphasizing is that this is an area that never had nets in the past, and there is therefore no long tradition of using LLIN, and yet people who have adequate access seem to be using them. Apart from that, by comparison to other studies, lower gender disparity in LLIN use (female vs male = 65.9 vs 63.9) was observed in the study population [12, 33, 34].

Literacy of head of the households, type of house, and household size were among the key determinants of LLIN use in the study population. In particular, children under 14 years of age, households living in pakka/khaprel (cemented house with tiled roof) houses with family size ≤ 4 members were more likely to use LLINs in the surveyed population (Table 3).

Odds of using LLINs were 0.67 times higher among the population residing in pakka type of houses as compared to those living in hut type of house structures. Sleeping arrangement and house structures are major determinants for LLIN use, possibly due to inadequate space and suitable places for hanging the nets in the hut type of houses [35, 36]. Similarly, odds of using LLIN increased in families with decrease in family size in the study area. In addition, higher education level of the head of the household was significantly associated with increase in odds of sleeping under a LLIN (OR, 95% CI = 1.2, 1.04–1.4; p = 0.012). Similar findings were reported by Ntuku et al. [13]. Likewise, larger households found to have lower net use was also reported in African studies [14–17].

After 1 year of distribution, fabric integrity was retained in 89% of total observed LLINs. A recent field study has reported durability of LLINs as of 2–3 years and after 2 years, more than half of LLINs usually fall in ‘replacement category’ [37].

Major behavioural determinants of non-use of LLIN were use of alternative mosquito control methods (14%), low mosquito density (6.8%), discomfort due to LLIN or not habitual of sleeping under the LLIN (6.8%) apart from non-availability of LLINs (46.1%). Similar determinants were also reported by earlier studies [18, 38, 39].

Despite high LLIN coverage (98.4%), intra-household availability was lower; hence, additional distribution of LLINs in the study area was required. The current study has highlighted the call for of regular assessment of LLIN use, top-up LLIN distribution and concurrent awareness about use of LLIN by all age groups for equitable protection against malaria in the population living in endemic areas.

## Limitations

This study has not assessed LLIN fabric integrity and survivorship. Hence, further follow-up studies on LLIN fabric integrity and survivorship is required to assess the net serviceable life and predict the timing of next round of LLIN distribution to maintain the adequate coverage at intra-household level.

## Conclusions

Regular awareness and maintenance of universal coverage is necessary to sustain the optimal LLIN coverage and usages in the population especially in high risk groups such as children under 5 years of age and evaluation of LLIN attrition in the malaria endemic areas. Health education and awareness about benefit of continuous use of LLIN should not be limited to only high risk groups but all household members to achieve the impact of this intervention at the community level. Further, top up distribution of additional LLINs is required where initial coverage is not achieved as per the revised norm of one net per two persons. The key reason for non-use of nets remains lack of access to LLINs. This underscores the importance of mass distributions achieving universal coverage in each household, not just overall coverage.

## Abbreviations

LLINs: long-lasting insecticidal nets
IRS: indoor residual spraying
MIS: Malaria Indicator Survey
RBM: Roll Back Malaria
NVBDCP: National Vector Borne Disease Control Programme
IIR: implications of insecticide resistance
PHC: Primary Health Centres
HHs: households
WHO: World Health Organization
SD: standard deviation
GEE: generalized estimating equation
